# Evidence of West Nile virus infection in migratory and resident wild birds in west coast of peninsular Malaysia

**DOI:** 10.1016/j.onehlt.2020.100134

**Published:** 2020-04-19

**Authors:** Mohd Yuseri Ain-Najwa, Abd Rahaman Yasmin, Abdul Rahman Omar, Siti Suri Arshad, Jalila Abu, Hussni O. Mohammed, Kiven Kumar, Shih Keng Loong, Jeffrine J. Rovie-Ryan, Ahmad-Khusaini Mohd-Kharip-Shah

**Affiliations:** a Department of Veterinary Laboratory Diagnostics, Faculty of Veterinary Medicine, Universiti Putra Malaysia, Malaysia; b Department of Veterinary Clinical Studies, Faculty of Veterinary Medicine, Universiti Putra Malaysia, Malaysia; c Department of Veterinary Pathology and Microbiology, Faculty of Veterinary Medicine, Universiti Putra Malaysia, Malaysia; d Laboratory of Vaccines and Immunotherapeutics, Institute of Bioscience, Universiti Putra Malaysia, Malaysia; e College of Veterinary Medicine, Department of Population Medicine and Diagnostic Sciences, Cornell University, USA; f Tropical Infectious Diseases Research & Education Centre, High Impact Research Building, University of Malaya, Malaysia; g Wildlife Conservation Division, Department of Wildlife and National Parks, Malaysia

**Keywords:** West Nile virus, Migratory, Resident, Wild bird, c-ELISA, RT-PCR

## Abstract

West Nile virus (WNV) is a zoonotic mosquito-borne flavivirus that is harbored and amplified by wild birds via the enzootic transmission cycle. Wide range of hosts are found to be susceptible to WNV infection including mammals, amphibians and reptiles across the world. Several studies have demonstrated that WNV was present in the Malaysian *Orang Asli* and captive birds. However, no data are available on the WNV prevalence in wild birds found in Malaysia. Therefore this study was conducted to determine the serological and molecular prevalence of WNV in wild birds in selected areas in the West Coast of Peninsular Malaysia. Two types of wild birds were screened, namely migratory and resident birds in order to explore any possibility of WNV transmission from the migratory birds to the resident birds. Thus, a cross-sectional study was conducted at the migratory birds sanctuary located in Kuala Gula, Perak and Kapar, Selangor by catching 163 migratory birds, and 97 resident birds from Kuala Gula and Parit Buntar, Perak at different time between 2016 and 2017 (Total, *n* = 260). Blood and oropharyngeal swabs were collected for serological and molecular analysis, respectively. Serum were screened for WNV antibodies using a commercial competitive ELISA (c-ELISA) (ID Screen® West Nile Competition Multi-species ELISA, ID VET, Montpellier, France) and cross-reactivity towards Japanese Encephalitis virus (JEV) was also carried out using the JEV-double antigen sandwich (DAS) ELISA. Oropharyngeal swabs were subjected to one-step RT-PCR to detect WNV RNA, in which positive reactions were subsequently sequenced. WNV seropositive rate of 18.71% (29/155) at 95% CI (0.131 to 0.260) and molecular prevalence of 15.2% (16/105) at 95% CI (0.092 to 0.239) were demonstrated in migratory and resident wild birds found in West Coast Malaysia. Phylogenetic analyses of the 16 WNV isolates found in this study revealed that the local strains have 99% similarity to the strains from South Africa and were clustered under lineage 2. Evidence of WNV infection in resident and migratory birds were demonstrated in this study. As a summary, intervention between migratory birds, resident birds and mosquitoes might cause the introduction and maintenance of WNV in Malaysia, however the assumption could be further proven by studying the infection dynamics in the mosquitoes present in the studied areas.

## Highlights

• Exposure to and the potential circulation of WNV among wild birds in Malaysia were demonstrated for the first time.
• The anti-WNV IgG antibodies prevalence of 18.71% and molecular WNV RNA prevalence of 15.2% were revealed in the tested wild bird cohort.
• This information has far-reaching public and animal health implications for Malaysia.

## 1 Introduction

West Nile virus (WNV) infection can cause significant morbidity and mortality in humans and animals worldwide, as were seen in the outbreaks in New York and India [4,23]. WNV was first reported in a woman from Uganda in 1937 [36]. Since then, the virus has spread globally and caused epidemic outbreaks on all continents except for Antarctica [31,36]. Transmission of the virus occurs in an enzootic cycle between wild birds and mosquitoes, in which wild birds serve as reservoirs and amplifiers, while mosquitoes such as *Culex* spp. and *Aedes* spp. act as vectors that transmit the virus to other mammalian, reptilian or amphibian hosts through salivary secretions during blood meals [4,7,9]. Following WNV infection, the virus titers are higher in birds compared to other animals, and although most infected birds are asymptomatic, the increased levels of viremia in birds facilitates WNV transmission to mosquitoes during blood-meal [27].

Emerging and re-emerging zoonotic diseases contracted from wildlife such as Nipah virus-related illness, Japanese encephalitis (JE), rabies and avian influenza are endemic in Malaysia [10,13,14,25]. Besides, mosquitoes-borne diseases namely dengue, JE, chikungunya, zika, getah virus disease and malaria are prevalent in Malaysia [1,5,30,34,40]. As for WNV, evidence of the infection in 1.21% (9/742) *Orang Asli* in several states of Peninsular Malaysia [17] and 4.41% (3/68) in companion bird populations in Selangor [28] were demonstrated. Additionally, the Kunjin virus which is a WNV sub-type that is endemic in Australia was detected in Sarawak in 1970 from *Culex pseudovishnui* mosquitoes [6,20].

Being a hot and humid country, Malaysia provides the perfect environment for mosquitoes to flourish and thrive. The *Culex* spp. and *Aedes* spp. of mosquitoes, known to be vectors of several tropical vector-borne diseases, are found widespread in Malaysia. The prevalence of WNV among wild birds has, until now, not been investigated in Malaysia. Since wild birds play a major role in WNV transmission and WNV is pathogenic to humans and animals, this study was carried out to determine the prevalence of WNV in wild birds in the West Coast of Peninsular Malaysia. Furthermore, several studies have suggested the theory of “migrant bird as the introductory host” of WNV, and therefore the present study was conducted in two types of wild birds namely migratory and non-migratory (resident) birds to assess the possibility of the transmission of WNV from migratory birds to resident birds found in Malaysia.

## 2 Materials and methods

### 2.1 Ethical and permit approval

All experimental procedures were conducted following guidelines approved by the Institutional Animal Care and Use Committee (IACUC) of Universiti Putra Malaysia (UPM) with the reference number UPM/IACUC/AUP NO: R043/17. The sampling permit was also approved and granted by the Department of Wildlife and National Parks (DWNP), Peninsular Malaysia with the research permit number JPHL&TN (IP): 100–6/1/14.

### 2.2 Sample collection

A cross-sectional study was conducted to determine the prevalence of WNV infection in wild birds in selected areas at the West Coast of Peninsular Malaysia. Birds owned as pets were excluded from this study. Study sites were selected based on the areas where the migratory birds were commonly seen in West Coast Malaysia. The migratory birds were caught at migratory bird sanctuaries located in Kuala Gula, Perak (4.933^0^N, 100.467°E) and Kapar, Selangor (3.1373^0^N, 100.3744°E). Kuala Gula is located in the Perak state, an area with paddy cultivation and presence of mangroves. Meanwhile, Kapar is located in the Selangor state, where houses of electricity generating power plants are found and is surrounded by inundated water reservoirs. On the other hand, resident wild birds were sampled in the Perak state only, namely Kuala Gula and Parit Buntar (5.1474^0^N, 100.4212°E), where these birds have acclimated to living close to human residential areas. The birds were trapped using mist and hand nets. As WNV is a zoonotic virus, the sampling was carried out by trained personnel with appropriate personal protective equipment according to biosafety guidelines. Sample collection was performed based on convenient sampling and was conducted in February 2016, May 2016 and October 2017, to coincide with the migratory birds landing period in Malaysia.

A total of 260 wild birds (*n* = 260) were caught and blood was drawn from the jugular or brachial vein for serological analysis. While, oropharyngeal swabs were taken for molecular analysis. However, due to the limitations of this study, not all birds had their serum and swab samples taken. Table 1 shows the number of wild birds sampled in this study based on the category, family, species, states and type of samples.

**Table 1:** Number of wild birds sampled based on category, family, species, states and types of samples.

Category	Family	Species	States	Number of wild birds	Total	Total serum samples obtained	Total oropharyngeal swabs samples obtained
Migratory	*Charadriidae*	Lesser Sand Plover	Selangor	27	163	155	105
			Perak	5			
		Greater Sand Plover	Selangor	17			
			Perak	7			
	*Scolopacidae*	Terek Sandpiper	Selangor	23			
		Common Redshank	Selangor	28			
			Perak	20			
		Common Sandpiper	Selangor	32			
		Whimbrel	Perak	2			
	*Alcedinidae*	Black-capped Kingfisher	Perak	2			
Resident	*Ardeidae*	Little Egret	Perak	85	97		
		Cattle Egret		1			
		Black Crowned Night Heron		8			
		Striated Heron		2			
	*Charadriidae*	Red-wattled Lapwing	Selangor	1			

Overall Total = 260 birds

Note: The table shows the total number of birds sampled between February 2016 and October 2017 by using mist net and hand net trapping. The total number of samples were inconsistent with the total number of birds due to not all serum and oropharyngeal swabs could be obtained from each bird at the same time particularly due to the size of the birds.

### 2.3 Serological analysis

#### 2.3.1 Serum processing

Blood was collected from the jugular or brachial vein of wild birds and placed into EDTA tubes (BD Vacutainer®, New Jersey, USA). Serum samples were collected from blood after clarified at 252 x *g* (Thermo Fisher Scientific, Waltham, USA) for 10 min. All procedures were carried out in the class II, type A2 biosafety cabinet (Esco (M), Singapore). The labeled serum samples were stored at -80 °C (SANYO Ultra Low, Osaka, Japan) until further analysis.

#### 2.3.2 IgG based WNV Competitive-ELISA (c-ELISA)

Serum samples were tested for WNV antibodies using commercial c-ELISA test kit (ID Screen West Nile Competition Multi-species ELISA, ID VET, Montpellier, France) according to the manufacturer's instructions. The kit was developed to detect IgG antibodies directed against the envelope protein (prE) of WNV. The prE monoclonal antibody used in the c-ELISA cross-reacts with antibodies against other members of the Flavivirus family namely, the Japanese encephalitis virus (JEV) and the tick-borne encephalitis virus (TBEV). Since the latter is not endemic in Malaysia, positive WNV serums were assessed for cross reactivity against JEV using specific double-antibody sandwich ELISA (DAS-ELISA) (Sun red, Shanghai, China) pre-coated with anti-JEV antibodies.

#### 2.3.3 Data analysis

In the c-ELISA analysis, the positive and negative controls were added in duplicate in each test and if the sample shows S/N% (S = sample OD; N = negative control OD) less than or equal to 40%, it was then viewed as positive. While for DAS-ELISA, the positive and negative controls were added in duplicate in each test, and sample showing OD values that were greater than the equal cut-off value, was considered as positive. The prevalence of WNV antibodies in this study was calculated as a percentage of positive samples over the total number of examined samples at 95% confidence interval. Data obtained in this study could not be further analyzed statistically for risk factors because of the uneven ratio of the groups that could create potential bias.

### 2.4 Molecular analysis

#### 2.4.1 Sample processing, and RNA extraction

The oropharyngeal swabs were collected using sterile cotton swabs and then were kept in sterile tubes contain 2 mL of phosphate buffered saline (PBS) at pH 7.4. Total RNA was extracted from the swab using TRIzol reagent (TRIsure, Bioline, London, UK) according to the manufacturer's instructions. The concentration and purity of the extracted RNA were measured using the BioPhotometer (Eppendorf, Hamburg, Germany) at the wavelength of 260/280 nm.

#### 2.4.2 Plasmid and primers

The synthetic gene (plasmid) was used as positive control in this study, targeted a highly conserved region of the WNV genome between the capsid (C) and the pre-membrane (prM) protein. The plasmid and primer sets for RT-PCR were designed based on selected 15 WNV strains (Accession Nos. KU978767, DQ318019, AY532665, EF429200, EF429197, KP789953, KT359349, KT757322, KJ883346, KT757319, KT757323, EU068667, HM147822, DQ176636 and KC131126) using Molecular Evolutionary Genetics Analysis (MEGA) software version 7. Sequences of the primers used in this study were as follows: forward primer (5′- CCAATACGTTTCGTGTTGG −3′); and reverse primer (5′- GGAAATGACCCTGAAGACAT -3′).

#### 2.4.3 RT-PCR assay

The extracted RNA from the oropharyngeal samples were further subjected to one-step RT-PCR using MyTaq One-Step RT-PCR (Bioline, Memphis, USA) in a 25 μL reaction with primers (10 μM) specific for WNV. Nucleic acid amplification was performed on a Mastercycler gradient thermal cycler (Eppendorf, Hamburg, Germany) with the following protocol: reverse transcription step for 20 min at 45 °C, then polymerase activation at 95 °C for 1 min followed by 40 cycles of 10 s denaturation at 95 °C, 10 s annealing at 52 °C and 30 s extension at 72 °C. The final extension had similar conditions as the extension step except that it took 5 min for the final extension period. The amplification volume was performed according to the manufacturer's instruction, with minor modifications in which the primer volume was reduced from 1.0 μL to 0.5 μL to reduce the formation of primer dimers. The PCR product was analyzed on a 1.5% agarose gel with TAE (Tris-acetate-EDTA) running buffer and stained with GelRed™ Nucleic Acid Gel Stain (Biotium, California, USA) for the visualization of amplified RT-PCR products. The PCR reaction was considered positive when a 470-bp fragment containing the portion of the C and the prM gene, was amplified and aligned with a positive control band.

#### 2.4.4 Bioinformatics analysis of WNV partial sequences

Positive RT-PCR product was purified using the Nucleospin Gel and PCR Clean-up kit (Macherey-Nagel, Düren, Germany) and subsequently subjected to DNA sequencing using the ABI PRISM 3730xl Genetic Analyzer (Applied Biosystems, California, USA). The nucleotide sequences obtained from the sequencing analysis were compared using the Basic Local Alignment Search Tool (BLAST) algorithm. Multiple alignments of the WNV nucleotide sequences were carried out using Clustal Omega Version 2. The 16 partial coding sequences of WNV were used to construct a phylogenetic tree using the Molecular Evolutionary Genetics Analysis (MEGA) 7 software. Sequences obtained from this study were compared to 36 other isolates from different WNV complete genomes available in the GenBank (Accession Nos. EF429197.1, DQ211652.1, AF196835.2, AY765264.1, AY277251.1, DQ256376.1, KC601756.1, AF404756.1, EU082200.2, KM203862.1, MF984352.1, KU588135.1, JX442279.1, HM152773.1, AY688948.1, DQ786573.1, KT359349.1, AF404757.1, KJ934710.1, KJ577738.1, DQ176636.2, MH021189.1, AY701412.1, KX394398.1, AY262283.1, HM152775.1, AY268133.1, AY712945.1, AY660002.1, AY603654.1, AY490240, EF429200.1, DQ318020.1, AY532665.1, JX123030.1 and KT934803.1). The resulting nucleotide sequence information was analyzed using the neighbor-joining method [29] and the evolutionary distances were computed using the Maximum Composite Likelihood method [37].

## 3 Results

### 3.1 Seroprevalence of WNV in wild birds

Out of 155 serum samples analyzed using WNV c-ELISA, 30 samples showed S/N% value less than or equal to 40%, which was considered as positive reaction. All of these serum samples were further analyzed using JEV DAS-ELISA and only one sample showed positive reaction. Therefore, this study found positive reaction for anti-WNV IgG antibodies in 18.71% (29/155) of the tested serum at 95% CI (0.131 to 0.260). Table 2 shows the distribution of WNV antibody positive wild birds according to location, category, family and species.

**Table 2:** WNV antibodies detection according to sampling states, category, family and species of wild birds.

States	Category of wild birds	Family of wild birds	Species of wild birds	Number of wild birds for c-ELISA analysis	c-ELISA positive	Number of wild birds for DAS-ELISA analysis	DAS-ELISA positive
Perak	Migratory	*Charadriidae*	Lesser Sand Plover	2	0	0	0
			Greater Sand Plover	3	0	0	0
		*Scolopacidae*	Common Redshank	8	0	0	0
			Whimbrel	1	0	0	0
		*Alcedinidae*	Black-capped Kingfisher	1	0	0	0
	Resident	*Ardeidae*	Little Egret	48	1	2	1
			Cattle Egret	1	0	0	0
			Black Crowned Night Heron	8	0	0	0
			Striated Heron	1	1	1	0
Selangor	Migratory	*Charadriidae*	Lesser Sand Plover	19	9	9	0
			Greater Sand Plover	9	2	2	0
		*Scolopacidae*	Terek Sandpiper	16	5	5	0
			Common Redshank	23	11	11	0
			Common Sandpiper	14	0	0	0
	Resident	*Charadriidae*	Red-wattled Lapwing	1	0	0	0
Overall Total				155	29	30	1

c-ELISA – competitive ELISA; DAS-ELISA – double-antibody sandwich ELISA.

**Fig. 1:**
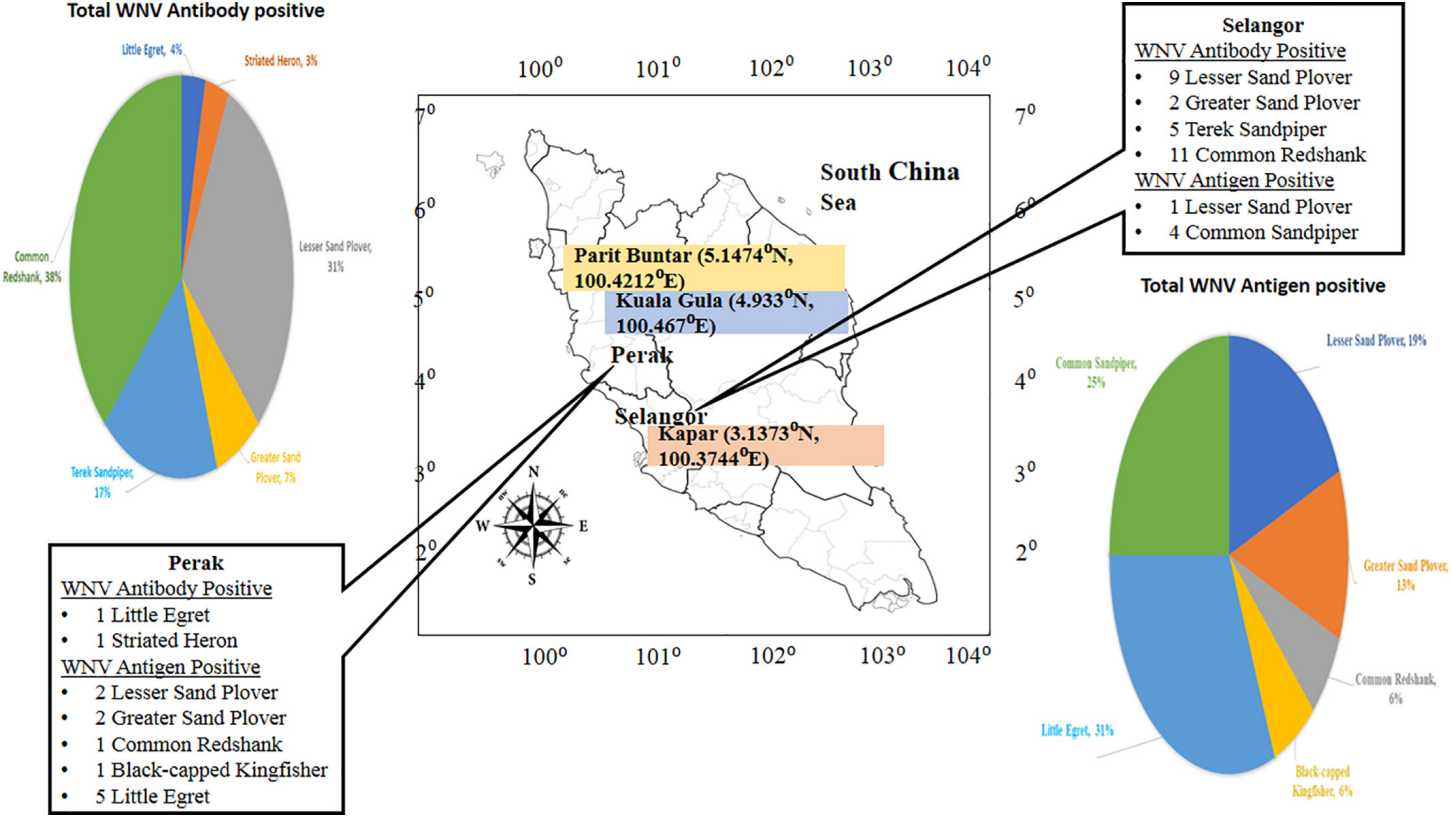
Geographical Distribution of Positive WNV Serology and RNA according to wild bird species in West Coast Peninsular Malaysia.

Only two wild birds were WNV seropositive in Perak which came from the resident wild bird. These birds are from the species of Little Egret and Striated Heron, in which both are from the *Ardeidae* family. At Selangor, all 27 of the WNV seropositive wild birds were from migratory birds. Among the species found were Common Redshank (11 positive), Terek Sandpiper (5 positive), Lesser Sand Plover (9 positive) and Greater Sand Plover (2 positive). The first two are from the *Scolopacidae* family while the rest are from the *Charadriidae* family. None of the migratory birds were seropositive in Perak. And because none of the resident birds were sampled in Selangor, therefore the data is not available. Fig. 1 shows the geographical and species distribution of the WNV seropositivity in detail.

### 3.2 Molecular prevalence of WNV in wild birds

**Fig. 2:**
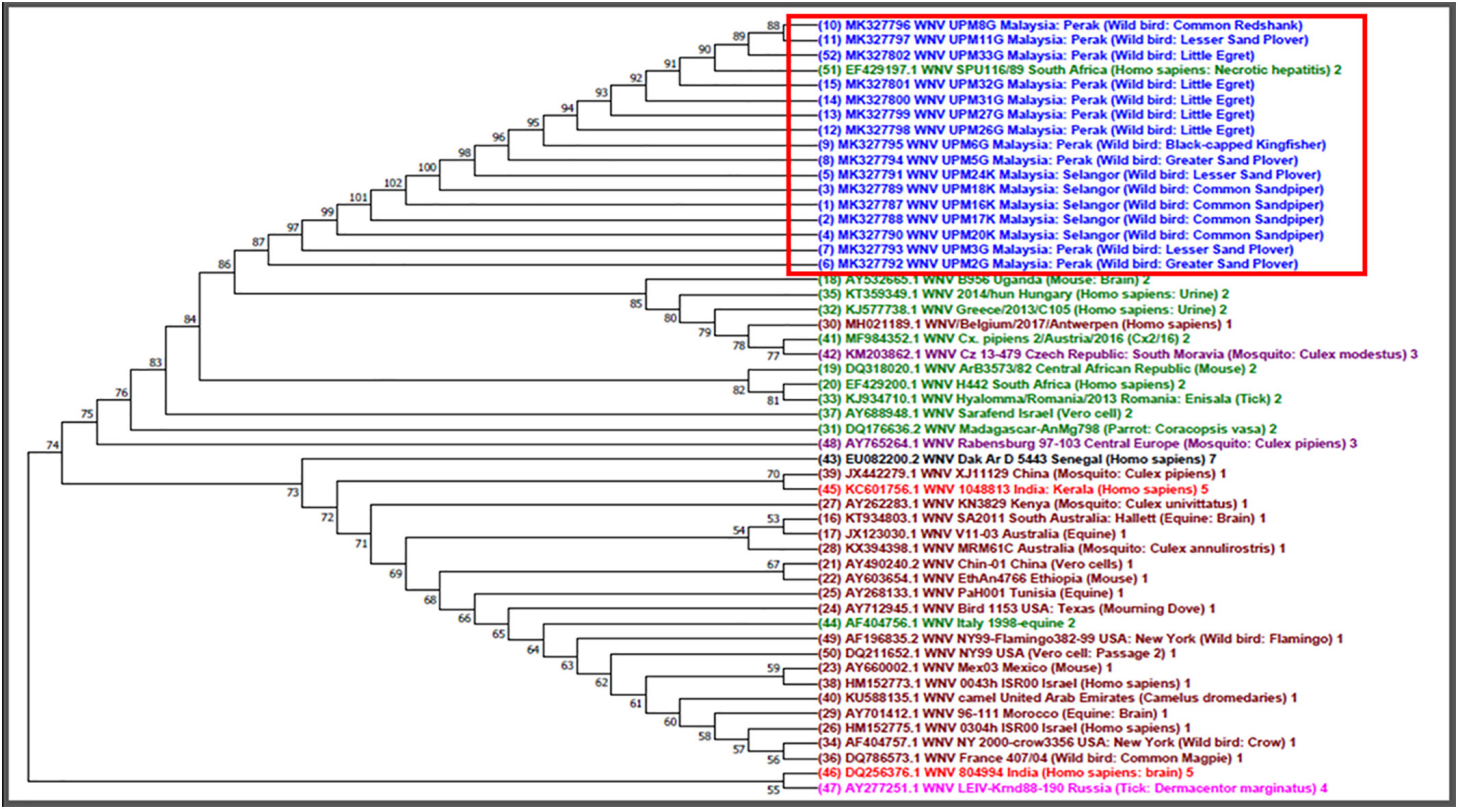
Evolutionary relationships of taxa in WNV of wild birds. The analysis involved 52 nucleotide sequences including 16 sequences (in the red box) which have 470-bp nucleotide length obtained from wild birds in this study. (For interpretation of the references to colour in this figure legend, the reader is referred to the web version of this article.)

One step RT-PCR revealed that 15.2% (16/105; 95% CI; 0.092 to 0.239) of the tested wild birds were positive for WNV antigen. Positive detection of WNV RNA was detected in both Perak and Selangor in migratory birds while resident birds were positive in Perak only as no resident birds were sampled in Selangor (Table 3). Fig. 1 shows the distribution of the WNV antigen positive wild birds according to species and location. Among the species of the resident birds that were positive came from *Ardeidae* family was Little Egret (5 positive), followed by migratory birds namely from *Charadriidae* family (Lesser Sand Plover 2 positive, Greater Sand Plover 2 positive), *Scolopacidae* family (Common redshank 1 positive) and *Alcedinidae* family (Black-capped Kingfisher 1 positive). Amplicons of RT-PCR positive samples were further sequenced and the sequences were deposited into GenBank under the following accession numbers: MK327787-MK327802. A phylogenetic tree was constructed with these isolates together with 36 other WNV sequences available in GenBank (Fig. 2). The WNV sequences found in the current study were grouped together with the WNV lineage 2 isolate (Accession No. EF429197.1).

**Table 3:** WNV RNA detection according to sampling states, category, family and species of wild birds.

States	Category of wild birds	Family of wild birds	Species of wild birds	Number of wild birds for RT-PCR analysis	RT-PCR positive
Perak	Migratory	*Charadriidae*	Lesser Sand Plover	3	2
			Greater Sand Plover	4	2
		*Scolopacidae*	Common Redshank	12	1
			Whimbrel	1	0
		*Alcedinidae*	Black-capped Kingfisher	1	1
	Resident	*Ardeidae*	Little Egret	37	5
			Striated Heron	1	0
Selangor	Migratory	*Charadriidae*	Lesser Sand Plover	8	1
			Greater Sand Plover	8	0
		*Scolopacidae*	Terek Sandpiper	7	0
			Common Redshank	5	0
			Common Sandpiper	18	4
Overall Total				105	16

RT-PCR – reverse transcriptase PCR.

## 4 Discussion

West Nile Virus (WNV) is an arbovirus transmitted by mosquitoes primarily the *Culex* species. Mosquitoes usually acquired the virus from wild birds that served as the reservoir and amplifier of WNV during blood-feeding. Antibody against WNV has been found in Malaysian *Orang Asli* [17] and companion birds [28] suggesting that WNV is circulating in Malaysia [2]. As there is a lack of study on WNV in the wild birds found in Malaysia, this study was carried out to determine WNV serological and molecular prevalence in selected areas in the West Coast of Peninsular Malaysia. Our group also is conducting similar studies in other species of animals particularly macaque, bats, horse, ruminants, swine, cats, dogs as well as in mosquitoes (yet to be published) and a few preliminary works published in conference proceedings revealed that WNV antibodies and/or antigen were detected in macaque, bats, horse and swine [3,8,15,21,35,39].

Migratory birds are birds that move regularly according to the season along different flyway routes across the Northern and Southern hemisphere [38]. Migratory birds are capable of transmitting WNV to the new vectors and hosts located thousands of kilometers away, to as far as new continents along the major wild bird flyways across Asia, Africa and Europe [11,19,24]. While resident birds are non-migrant, non-captivated and present in the wild and some of these birds have acclimated to living close to human residential areas [33]. The evidence of WNV transmission in the non-migrant or resident birds are also widely demonstrated [12,30,32]. Thus, apart from screening WNV antibody and antigen, this study also wanted to explore any possibility whether the migratory birds have introduced the virus to the resident birds in Malaysia.

The location of the study was chosen based on the area where resident birds are present along with the common area where migratory birds usually land during the migration period in Malaysia. There are several locations where migratory birds are commonly seen in Malaysia and these locations are known as migratory bird sanctuary. Kuala Gula and Kapar are among the list and therefore was selected for the purpose of this study. In addition to that, Kuala Gula and Parit Buntar are also area where resident birds particularly water birds were hugely available.

Evidence of WNV exposure was determined by using serological assays namely WNV IgG based competitive ELISA. This assay has the tendency to cross-react with other Flavivirus particularly Dengue virus (DEV), JEV and Tick-borne virus (TBEV). As DEV and TBEV is not prevalent in birds and Malaysia, respectively, while JEV is endemic and prevalent in Malaysian animals including birds as well as in humans, therefore this study only incorporated further JEV based ELISA to examine any occurrence of cross-reactivity. A gold standard test to confirm positive WNV antibody is neutralization test [22], however this study did not perform the test due to a few limitations. Having said that, WNV IgG based ELISA is a reliable marker in screening presence of WNV neutralizing antibodies when vaccination and infection of WNV are absent in the past. Therefore, the usage of the kit fit the purpose of this study. Out of 155 bird's serum tested for WNV IgG based ELISA, 30 birds showed seropositivity and among these birds, only one showed cross-reactivity to JEV and this bird is excluded from the WNV prevalence report. Seroprevalence of WNV in wild birds in this study is 18.71% (29/155) in which 27 were from migratory birds while 2 were resident water birds. Most of the seropositive migratory birds belong to the family of *Scolopacidae*. A separate study in Germany also reported the detection of WNV antibodies from the *Scolopacidae* family [32].

On the other hand, one step RT-PCR was carried out to determine presence of WNV shedding from oropharyngeal swabs of the wild birds found in West Coast Malaysia. The PCR reaction revealed that 15.2% (16/105) of the wild birds in this study were positive for WNV antigen. The positive birds were from the *Charadriidae*, *Scolopacidae*, *Alcedinidae* and *Ardeidae* families. Similar to serological detection, most of the positive birds belonged to the migratory birds (11/105) and the rest were from resident birds, particularly Little Egret. However, high seropositivity and WNV RNA positive in migratory birds were expected due to the uneven number of the resident and migratory birds sampled in this study. Based on nucleotide sequence analyses, the 16 isolates showed 99% similarity to the SPU116/89 strain which originated from South Africa. Among the seven lineages of WNV, isolates found in this study were belong to the WNV lineage 2, originating from a human who succumbed to necrotic hepatitis in South Africa [18].

The present study revealed that birds that were positive for WNV RNA were clinically healthy and seronegative towards WNV antibody. Seropositive birds based on IgG assay suggested that the birds were exposed to WNV in the past. Study by Komar et al. 2003 [11], demonstrated that experimental infection of WNV in wild birds showed shedding of the WNV after oral WNV inoculation was observed until day 10th post infection. This finding suggested that WNV antigen positive birds from this study might be exposed to WNV recently as opposed to the sampling time. As this study showed resident birds were also positive towards WNV antibody and WNV antigen, therefore the probability of WNV dissemination are possible and might be attributed by several factors. During feeding at the water body, migratory birds might transmit WNV to other resident birds through the oral-fecal route [26]. In addition, the ornithophilic characteristics of *Culex* spp., which are also the vectors of WNV, increases the likelihood of contact with wild birds, thereby also enhancing the possibility of WNV transmission to resident birds [16]. Migratory birds, resident birds and mosquitoes intervention might cause the introduction and maintenance of the WNV in Malaysia.

In summary, findings from the current study indicated that WNV is circulating among the local wild birds, as demonstrated by the detection of WNV antibodies and antigen. Evidence of WNV in Malaysia is attributable to several factors; among them, the presence of migratory birds as the main reservoir of the virus, the presence of breeding sites for mosquitoes that serve as vectors, as well as the ability of the virus to infect a wide range of susceptible hosts. Although there is yet to be any clinical report of WNV infections and disease outbreak in humans or animals, this study further substantiated WNV circulation in Peninsular Malaysia. Therefore, it is imperative to implement a WNV monitoring program with the aim of preventing the transmission of WNV. In order to achieve the One Health aspiration, further emphasis must be directed on the reduction of vector breeding sites, as well as public education and awareness on mosquito control. Following this study, the Department of Wildlife Malaysia along with the cooperation of the Department of Veterinary Service Malaysia had included WNV in the routine bio surveillance in wild birds apart from avian influenza virus and JEV.

## Funding information

This research was supported by 10.13039/501100004530Universiti Putra Malaysia (UPM) under the research grants UPM 700-2/1/GP-IPM/2016/9510500 and UPM 700-2/1/GP-IPS/2017/9547800.

## Declaration of Competing Interest

The authors declare no competing interests.
